# Characterization of Efficiency and Mechanisms of Cold Atmospheric Pressure Plasma Decontamination of Seeds for Sprout Production

**DOI:** 10.3389/fmicb.2018.03164

**Published:** 2018-12-19

**Authors:** Alexandra Waskow, Julian Betschart, Denis Butscher, Gina Oberbossel, Denise Klöti, Annette Büttner-Mainik, Jozef Adamcik, Philipp Rudolf von Rohr, Markus Schuppler

**Affiliations:** ^1^Institute of Food, Nutrition and Health, ETH Zurich, Zurich, Switzerland; ^2^Institute of Process Engineering, ETH Zurich, Zurich, Switzerland; ^3^Seed Quality, Agroscope, Zurich, Switzerland

**Keywords:** cold atmospheric pressure plasma, seed decontamination, sprout production, atomic force micorscopy (AFM), dielectric coplanar surface barrier discharge

## Abstract

The consumption of fresh fruit and vegetable products has strongly increased during the past few decades. However, inherent to all minimally processed products is the short shelf life, and the risk of foodborne diseases, which have been increasingly related to such products in many parts of the world. Because of the favorable conditions for the growth of bacteria during the germination of seeds, sprouts are a frequent source for pathogenic bacteria, thus highlighting the need for seed decontamination to reduce the risk of foodborne illness. Consequently, this study focused on cold atmospheric pressure plasma (CAPP) treatment of artificially inoculated seeds in a diffuse coplanar surface barrier discharge to determine the inactivation efficiency for relevant foodborne pathogens and fungal spores. Plasma treatment of seeds resulted in a highly efficient reduction of microorganisms on the seed surface, while preserving the germination properties of seeds, at least for moderate treatment times. To characterize the mechanisms that contribute to microbial inactivation during plasma treatment, an experimental setup was developed to separate ultraviolet light (UV) and other plasma components. The combination of bacterial viability staining with confocal laser scanning microscopy was used to investigate the impact of ozone and other reactive species on the bacterial cells in comparison to UV. Further characterization of the effect of CAPP on bacterial cells by atomic force microscopy imaging of the same *Escherichia coli* cells before and after treatment revealed an increase in the surface roughness of treated *E. coli* cells and a decrease in the average height of the cells, which suggests physical damage to the cell envelope. In conclusion, CAPP shows potential for use as a decontamination technology in the production process of sprouts, which may contribute to food safety and prolonged shelf life of the product.

## Introduction

Increased consumption, large scale production and more efficient distribution of fresh produce have contributed to an increase in the number of illness outbreaks caused by this commodity over the past two decades (Olaimat and Holley, 2012). In particular, sprouts have gained worldwide popularity due to their nutritional values and health benefits (Kylen and Mccready, 1975; Kocharunchitt et al., 2009). However, despite the healthy image associated with sprouts, they represent one of the most common vehicles for produce-associated bacterial foodborne illnesses, and have been identified as the primary source of pathogens in many outbreaks (Sikin et al., 2013). A variety of foodborne pathogens such as pathogenic *Escherichia coli, Salmonella, Listeria monocytogenes*, and *Bacillus cereus* are frequently isolated from sprouted seeds and documented as causative agents of outbreaks of foodborne illness associated with sprouts (NACMCF, 1999). The key aspect that increases the risk of foodborne disease from sprouts compared to other fresh produce is the rapid growth of the bacteria due to the favorable conditions during the sprouting process such as high water activity, warm temperature, neutral pH, and availability of nutrients (Prokopowich and Blank, 1991). Therefore, decontamination of seeds is requested by the National Advisory Committee on Microbiological Criteria for Foods (NACMCF, 1999). Many sprout manufacturers apply 20,000 ppm of calcium hypochlorite solution to seeds before germination as recommended by the U.S. Food and Drug Administration (FDA). However, chlorine-based treatment achieves on average a reduction of 1–3 log CFU/g only and is associated with negative health effects and environmental issues (Sikin et al., 2013). Furthermore, in Switzerland and many EU countries application of chlorine as wash-based disinfectant for food production is strictly restricted due to the release of excessive amounts of potentially harmful disinfection by-products in the water (Van Haute et al., 2013).

In Japan, heat treatment represents the most common decontamination method for mung bean seeds (Bari et al., 2010). Although treatment for 5 min at 57°C or 60°C reduced *Salmonella* to <1 CFU/g, without substantial loss of germination properties, slightly higher temperatures and prolonged treatment times caused significant declines in seed germination (Jaquette et al., 1996). This narrow temperature range between treatment efficacy and seed injury makes it difficult to rely solely on heat for the elimination of pathogens. Consequently, most of the recent work in this area has involved the evaluation of combination treatments and the most successful strategy for adequate inactivation of microorganisms while maintaining germination properties is coupling heat with chlorine-based or organic sanitizers. However, such combinations turned out to not be sufficient for complete elimination of pathogens from different types of seeds (Ding et al., 2013). Although many methods have been even more effective than the FDA recommended method of 20,000 ppm hypochlorite, so far no treatment has been able to completely remove pathogens from sprout seeds while maintaining the germination capacity of the seeds (Montville and Schaffner, 2004; Bourke et al., 2018).

In conclusion, reliable methods for thorough decontamination of all types of seeds are still lacking and alternative intervention approaches have to be considered (Ding et al., 2013). Decontamination of seeds by cold atmospheric pressure plasma (CAPP) represents a promising alternative to conventional sterilization methods (Hertwig et al., 2018). Plasma is a fully or partly ionized gas, often referred to as the fourth state of matter. Besides plasmas in nature (e.g., sun, aurora borealis, lightning), there is also a large variety of technical applications ranging from fusion reactors with temperatures of more than 10^8∘^C to non-thermal plasma with gas temperatures below 100°C, which is also called non-equilibrium (non-thermal) or cold plasma. Such cold plasma has the unique feature of a low gas temperature, due to low-energy neutrals and ions, but high reactivity due to energetic electrons, which in turn generate a very reactive cocktail of active particles, such as charged particles, excited species, reactive neutrals and ultraviolet light (UV) photons that can cause detrimental effects on microorganisms (Hertwig et al., 2018). During the last decade, the effectiveness of cold plasma was demonstrated for the inactivation of a wide range of different microorganisms (Kayes et al., 2007; Selcuk et al., 2008; Misra et al., 2011; Tseng et al., 2012; Ziuzina et al., 2014; Butscher et al., 2016a,b). However, the precise inactivation mechanisms of cold plasma treatment and the contribution of the single components to the inactivation of microorganisms is still controversially discussed in literature (Lerouge et al., 2000; Boudam et al., 2006; Hertwig et al., 2018).

Non-thermal plasma processes have already been applied in many applications such as light sources, surface coatings, or ozone generation. In terms of food processing, non-thermal atmospheric plasma is a very recent technology (Knorr et al., 2011). However, as reviewed by Bourke et al. (2018), various studies about the application of CAPP for safe and sustainable food production were published during the last few years. The first studies on cold plasma treatment of plant seeds showed that the microbial load can be reduced while preserving the germination capacity of seeds and growth of sprouts (Selcuk et al., 2008; Bormashenko et al., 2012; Mitra et al., 2014; Butscher et al., 2016a). Moreover, several studies reported that cold plasma treatment of agricultural seeds has the potential to even improve the germination properties of the seeds and the growth parameters of seedlings (Randeniya and de Groot, 2015; Thirumdas et al., 2015; Butscher et al., 2016a).

In conclusion, the use of CAPP for controlling seed-borne microbial contamination seems to be a promising approach due to its benefits regarding efficient energy use, effective antimicrobial activity and seed health. Thus, the objectives in this study were the investigation of CAPP inactivation of relevant foodborne pathogens and fungi on lentil seeds in a diffuse coplanar surface barrier discharge (DCSBD) system, and the characterization of the microbial inactivation mechanisms by confocal laser scanning microscopy and atomic force microscopy (AFM) analysis of plasma treated bacteria.

## Materials and Methods

### Organism Strains and Culture Conditions

Strains of bacteria and fungi used in this study are listed in Table 1. All bacterial strains were maintained at -80°C in Brain-Heart-Infusion (BHI) broth (Biolife Italiana, Italy) supplemented with 20% glycerol. Working stocks were maintained on BHI agar (Biolife Italiana, Italy) at 4°C for a maximum of 1 month. Prior to each experiment, fresh overnight cultures were prepared by inoculating an isolated colony into 10 ml BHI broth (Biolife Italiana, Italy). Cultures were incubated for 16 h at 37°C with gentle shaking at 170 rpm. To verify growth of the bacteria, the optical density was measured using a Libra S22 (Biochrom; Cambridge, United Kingdom) spectrometer at 600 nm.

**Table 1:** Organisms used in this study.

Organism	Origin	Description
*E. coli* ATCC 8739	DSMZ, Germany	Strain Crooks, isolated from feces
*E. coli* B174 FAM 21843	Agroscope, Switzerland	Heat-tolerant Serovar O178:H12
*E. coli* B176 NCTC 12900	Agroscope, Switzerland	Serovar O157:H7, EHEC surrogate, non-STEC (stx-, eaeA+)
*L. monocytogenes* WS1001	Food Microbiology, ETH Zurich, Switzerland	Serovar 4b
*Salmonella enterica* DT7155	Food Microbiology, ETH Zurich, Switzerland	Serovar Typhimurium
*S. aureus* ATCC 25923	DSMZ, Germany	FDA, strain Seattle 1945, clinical isolate
*Geobacillus stearothermophilus*	Merck KGaA, Germany	Suspension of endospores
*Penicillium decumbens*	Food Microbiology, ETH Zurich, Switzerland	Isolated from food
*Aspergillus niger*	Food Microbiology, ETH Zurich, Switzerland	Isolated from food

*Aspergillus niger* and *Penicillium decumbens* were grown on YGC agar plates (Biolife Italiana, Italy) incubated at room temperature. After 18 days of growth in the dark, spores were harvested by pouring 10 ml of phosphate buffered saline (PBS) over the agar plate. The spores were agitated using a spatula to dissolve them in PBS and fungal hyphae and conidia were removed from the PBS solution by filtration through four layers of Mediset^®^ Faltkompressen (IVF Hartmann AG; Germany). To verify that hyphae and conidia were removed from the solution, the spore solution was investigated by phase contrast microscopy. Spores were quantified using a Helber chamber 0.02 mm (Ehartnack; Berlin, Germany) by counting four large squares of 5 × 5 small squares for each sample. The concentration of *Aspergillus niger* spores was determined to be on average 1.1 × 10^8^ spores per ml PBS, whereas the *Penicillium decumbens* spore solution contained on average 5.5 × 10^7^ spores per ml PBS.

### Inoculation of Lentil Seeds

For inoculation of lentil (*Lens culinaris*) seeds, 1 g of seeds was placed in a 15 ml centrifuge tube before adding 1 ml of the accordingly diluted bacterial overnight culture or spore solution, respectively. The seeds were inoculated by placing the tube for 5 min on a plate rotator and subsequently dried by placing in a sterile petri dish under a laminar flow bench for 4 h. During the drying process, the seeds were shaken once after 2 h of drying. After the drying process, the seeds were transferred to sterile 15 ml centrifuge tubes. For low initial inoculation level experiments, overnight cultures from *E. coli* B174, *L. monocytogenes, S. aureus* and *Salmonella* Typhimurium were further diluted by a factor of 1:1000.

### Treatment of Seeds in the Diffuse Coplanar Surface Barrier Discharge

Seed samples were treated in an atmospheric pressure DCSBD developed by Robust Plasma Systems (RPS400; Roplass s.r.o., Brno, Czechia), which is described in detail elsewhere (Šimor et al., 2002; Černák et al., 2011; Homola et al., 2012). The DCSBD consists of a high number of various microdischarges and creates a homogenous plasma layer with approximately effective 0.3 mm thickness on the top of the planar dielectric barrier which is a 96 mm × 230 mm alumina ceramic plate. The maximum nominal power directed into the plasma discharge was 400 W, which corresponded to maximum plasma intensity. Ozone and UV were measured by optical emission spectroscopy using an optical fiber (QP400-3-SR-BX, Ocean Optics) connected to a spectrometer (USB2000+XR1-ES).

Prior to the inactivation experiments, all parts in contact with seeds were sterilized using 70% ethanol. All experiments were conducted applying the DCSBD system in its original configuration at maximum power using ambient air as the operating gas. Static treatment was performed by placing dry lentil seeds equally distributed on the surface of the dielectric plate of the RPS400. After ignition of the plasma, the intensity was gradually increased to maximum by gradually adjusting the rotation swivel to 10 over a period of 30 s. The same procedure was repeated in reverse for turning off the plasma. Each side of the lentil seed was treated separately by flipping the lentil seeds after halftime using sterile tweezers. Thus, the total plasma treatment time for lentils corresponds to the total time spent on the plasma field. After plasma treatment, the seeds were placed in sterile 15 ml centrifuge tubes. For statistical purposes, all experiments were performed at least in triplicate. In each experiment, a control without plasma treatment was analyzed in parallel to calculate the logarithmic reduction of treated samples.

### Determination of Logarithmic Reduction

Logarithmic reduction of treated samples was determined by quantification of numbers of microorganisms (CFU/g seeds) after treatment using the standard plate count method. For this purpose, 9 ml PBS were added to 1 g of plasma treated seeds and 1 g of the untreated control. After transferring the seeds into stomacher bags with filter (Separator 400, Grade Products LTD; Coalville, United Kingdom), the samples were homogenized using a stomacher (Smasher^®^, AES chemunex; Geneva, Switzerland). A decimal dilution series in PBS was performed, before plating 100 μL of the suitable dilutions on regular (83 mm) selective agar plates. Selective media were used for *E. coli* (Chromocult Agar; Biolife Italiana, Italy), *Listeria monocytogenes* (Oxford Agar; Biolife Italiana, Italy), *Salmonella* Typhimurium (XLD Agar; Biolife Italiana, Italy), and *Staphylococcus aureus* (Baird-Parker Agar; Biolife Italiana, Italy). The dilution factor was dependent on the initial concentration and the treatment time. For low initial inoculation experiments, 1000 μl were plated on large (136 mm) agar plates to quantify microorganisms at the dilution of 10^-1^. After incubation of the agar plates, the number of colonies per plate was determined for regular agar plates containing 10 – 300 colonies and for large agar plates containing 10 – 500 colonies. An average was calculated from plates of two adjacent dilutions whenever possible. These values were used to calculate an average of colony forming units (CFU) per g of seeds. In order to determine and compare the performance of the DCSBD treatment, the logarithmic reduction was calculated as an indicator by how many log_10_ units the initial number of bacteria has been reduced by the treatment.

### Statistical Analyses

All experiments were performed at least three times. For statistical analyses of plating results, duplicates were averaged first, and mean as well as standard deviation was calculated from the three independent experimental results. As a reference, three control samples, which experienced identical sample preparation as the plasma treated seeds, were analyzed to determine the initial levels of microbial contamination. Finally, the logarithmic reduction in CFU is expressed by the difference in decimal logarithm of CFU/g for plasma treated seeds and the CFU/g determined for the untreated control samples.

### Evaluation of Germination Properties of Lentil Seeds After Plasma Treatment

The assessment of seed germination was performed with untreated control seeds and plasma treated seeds by the Agroscope, Seed Quality Laboratory, Zurich, Switzerland. Prior to the seed analysis, the statistically significant sample size of 66 seeds was calculated and the analysis was performed in triplicate. In brief, seeds were placed in pairs into pleated paper, soaked in 40 mL of tap water and kept at a germination temperature of 20°C at 85% humidity in a climate chamber with an 8 h day with light (Lux = 4000) and 16 h night cycle. Seeds and seedlings were inspected after 7 days. The presence of a root indicated normal germination, whereas in the case of an absence of a root, the seeds were classified as dead.

### Characterization of Microbial Inactivation Mechanisms

The antimicrobial effect of plasma could be the result of multiple inactivation mechanisms such as UV, temperature, electrons, ion bombardment and reactive species. In order to study the impact of plasma in more detail, an experimental setup was chosen that facilitated the separate investigation of UV and other plasma components. Plasma treatment of liquid suspensions of *E. coli* ATCC 8739 grown to exponential phase was performed in μ-Slide 2-well uncoated slides (ibidi; Planegg, Germany) in the RPS400 system. For selective UV treatment experiments, the slides were covered with a lid to prevent access of ozone and other reactive species to the *E. coli* cells. After 180 s treatment, aliquots of the bacterial suspensions were mixed with propidium iodide (Live/Dead BacLight Bacterial Viability Kit L13152, ThermoFischer Scientific) to determine membrane integrity or 50 mM 5-cyano-2,3-ditolyltetrazolium chloride (CTC; Fluka) for evaluation of metabolic activity. After incubation at room temperature, images of the samples were recorded using a confocal laser scanning microscope (Leica Microsystems GmbH; Wetzlar, Germany) equipped with a HCX PL FLUOTAR 100.0 1.30 oil objective. Analysis of images was performed using ImageJ Software (version 1.6.0, National Institutes of Health, United States). The percentage of bacteria showing fluorescence after plasma treatment was quantified from 10 randomly recorded images of each sample.

### Atomic Force Microscopy

For AFM analysis of plasma treated bacteria, 10 μl of an *E. coli* B174 overnight culture were transferred on a microscopic glass slide (76 mm × 26 mm × 1 mm; Menzel, Germany) and distributed on an area of approximately 40 × 20 mm using a cover slip before drying at room temperature for 15 min. CAPP treatment was performed on the DCSBD system for 60 s at 100% intensity by placing the microscopic slide with the side containing the bacteria facing the planar dielectric barrier. A distance of approximately 0.3 mm to the surface of the planar dielectric barrier of the DCSBD was adjusted by placing cover slips (18 mm × 18 mm, #1; Menzel, Germany) under both ends of the microscopic slide. For visualization and comparison of the surface of identical bacterial cells prior to and after plasma treatment, images were recorded from identical positions of the glass slide by AFM using Dimension FastScan scanning probe microscope (Bruker, United States) operating in tapping mode under ambient conditions. For further evaluation of AFM images, analysis of surface roughness was carried out and a height distribution data analysis was performed for viable, untreated *E. coli* cells and *E. coli* cells after 1 min CAPP treatment on the DCSBD system. Quantification of viable counts on glass slides was performed using standard plate count method. For this purpose, glass slides were transferred to 50 ml Falcon tubes containing 40 ml PBS and 20 g of glass beads (2 mm; VWR) and vortexed (3 × 15 s) before a decimal dilution series in PBS was performed. Plating of 100 μL of the suitable dilutions was performed on regular (83 mm) selective agar plates for *E. coli* (Chromocult Agar; Biolife Italiana, Italy). For statistical purposes, all experiments were performed at least in triplicate. In each experiment, a control (no plasma treatment) was analyzed in parallel to calculate the logarithmic reduction of the treated samples.

## Results

### Inactivation of *E. coli* on Lentil Seeds

**FIGURE 1:**
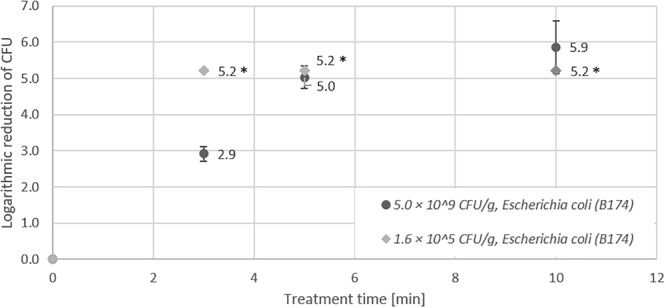
Effect of CAPP treatment of *E. coli* B174 on lentil seeds. Inoculation was performed either as high initial inoculation (5.0 × 10^9^ CFU/g) or low initial inoculation (1.6 × 10^5^ CFU/g) prior to treatment of seeds for 3, 5, or 10 min in the DCSBD. Logarithmic reductions were represented as log10 CFU/g ± standard deviation. The limit of detection was 10 CFU/sample for low initial inoculation and 100 CFU/sample for high initial inoculation. Logarithmic reduction below the limit of detection is indicated by an asterisk.

**FIGURE 2:**
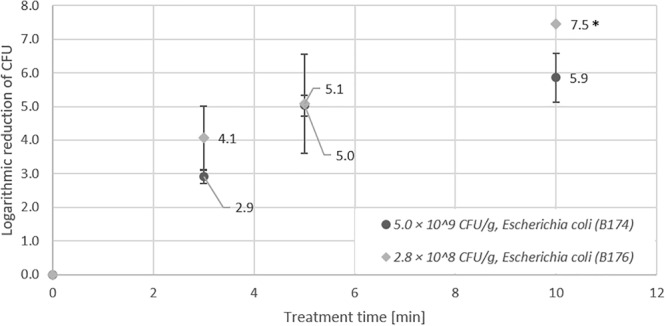
Effect of CAPP treatment of strains *E. coli* B174 and *E. coli B176* on lentil seeds. For both *E. coli* strains a high initial inoculation of lentil seeds was performed prior to treatment of seeds for 3, 5, and 10 min in the DCSBD. Logarithmic reductions were represented as log10 CFU/g ± standard deviation. The limit of detection was 100 CFU/g. Logarithmic reduction below the limit of detection is indicated by an asterisk.

In the first experiments, non-pathogenic *Escherichia coli* strains were used to establish the inoculation and inactivation procedure for plasma treatment of seeds in the DCSBD. In preliminary studies, we observed that a high portion of *E. coli* cells do not survive the drying process after artificial inoculation of samples. Therefore, we used the heat-tolerant strain *Escherichia coli* B174, due its higher resistance to desiccation as an important feature for the inoculation and drying procedure of seeds. In addition to high initial inoculation of seeds (5.0 × 10^9^ CFU/g) with *E. coli* B174, also low initial inoculation (1.6 × 10^5^ CFU/g) was performed to simulate more realistic contamination levels. For low initial inoculation samples, the detection limit (indicated by an asterisk) was already reached after 3 min (5.2 log CFU/g reduction), whereas for high initial inoculation, 5 min treatment was necessary to obtain an identical log reduction (Figure 1). The second *E. coli* strain (B176) used for CAPP inactivation experiments was a non-pathogenic surrogate for enterohemorrhagic *Escherichia coli* (EHEC). The maximum reduction determined for *E. coli* B176 with an initial inoculation of 2.8 × 10^8^ CFU/g was compared to the results obtained for *E. coli* B174. As shown in Figure 2, the EHEC surrogate revealed a higher logarithmic reduction compared to *E. coli* B174.

### Control of Foodborne Pathogens on Lentil Seeds

A major objective of this study was the determination of the inactivation efficiency for foodborne pathogens that are particularly relevant for sprout production because they are frequently linked to foodborne illnesses after the consumption of sprouts. In addition to the EHEC surrogate *E. coli* B176, lentil seeds were artificially inoculated with *Salmonella* Typhimurium, *Listeria monocytogenes* and *Staphylococcus aureus* and treated with CAPP in the DCSBD system. Also for these pathogens, the logarithmic reduction was determined for high (10^8^ – 10^9^ CFU/g) and low (10^4^ – 10^5^ CFU/g) initial inoculation on seeds.

**FIGURE 3:**
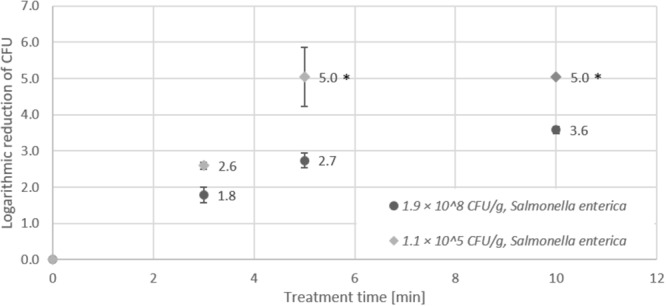
Effect of CAPP treatment of *Salmonella enterica* on lentil seeds. Inoculation was performed either as high initial inoculation (1.9 × 10^8^ CFU/g) or low initial inoculation (1.1 × 10^5^ CFU/g) prior to treatment of seeds for 3, 5 or 10 min in the DCSBD. Logarithmic reductions were represented as log10 CFU/g ± standard deviation. The limit of detection was 10 CFU/g for low initial inoculation and 100 CFU/g for high initial inoculation. Logarithmic reduction below the limit of detection is indicated by an asterisk.

Figure 3 shows that similar to *E. coli, Salmonella* Typhimurium revealed a much higher inactivation efficiency on seeds with low initial inoculation (5.0 log after 5 min) compared to the results for seeds with high initial inoculation (3.6 log after 10 min).

**FIGURE 4:**
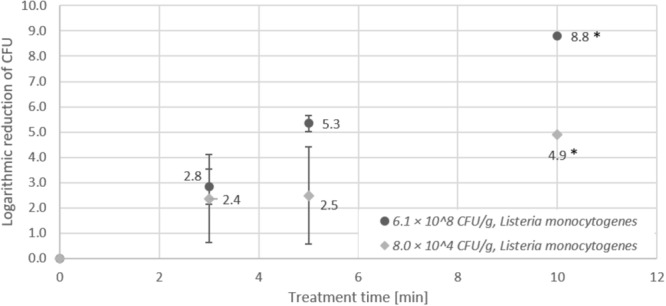
Effect of CAPP treatment of *Listeria monocytogenes* on lentil seeds. Inoculation was performed either as high initial inoculation (6.1 × 10^8^ CFU/g) or low initial inoculation (8.0 × 10^4^ CFU/g) prior to treatment of seeds for 3, 5, or 10 min in the DCSBD. Logarithmic reductions were represented as log10 CFU/g ± standard deviation. The limit of detection was 10 CFU/g for low initial inoculation and 100 CFU/g for high initial inoculation. Logarithmic reduction below the limit of detection is indicated by an asterisk.

Plasma treatment of seeds artificially contaminated with high (6.1 × 10^8^ CFU/g) and low (8 × 10^4^ CFU/g) numbers of *Listeria monocytogenes* (Figure 4) revealed the opposite trend. Logarithmic reduction (5.3 log after 5 min) was significantly higher for high than for low initial inoculation (2.5 log after 5 min). However, in both cases the detection limit was reached after 10 min treatment time, which corresponded to a maximum logarithmic reduction of 8.8 log for *Listeria monocytogenes*.

**FIGURE 5:**
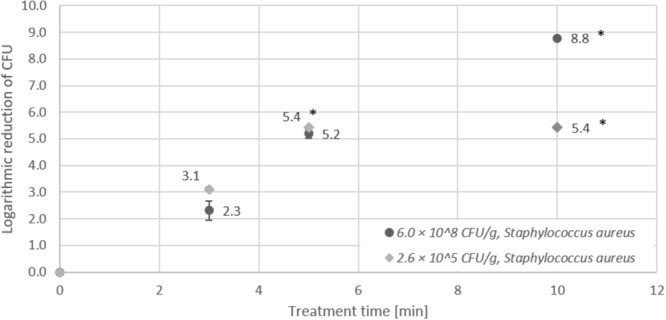
Effect of CAPP treatment of *Staphylococcus aureus* on lentil seeds. Inoculation was performed either as high initial inoculation (6.0 × 10^8^ CFU/g) or low initial inoculation (2.6 × 10^5^ CFU/g) prior to treatment of seeds for 3, 5, or 10 min in the DCSBD. Logarithmic reductions were represented as log10 CFU/g ± standard deviation. The limit of detection was 10 CFU/g for low initial inoculation and 100 CFU/g for high initial inoculation. Logarithmic reduction below the limit of detection is indicated by an asterisk.

In contrast to the results obtained for *Listeria monocytogenes* and *Salmonella* Typhimurium, CAPP treatment of *Staphylococcus aureus* on lentil seeds (Figure 5) revealed almost identical logarithmic reductions for high (6 × 10^8^ CFU/g) and low (2.6 × 10^5^ CFU/g) initial contamination of seeds. As obvious from Figure 5, the detection limit (<100 CFU/g) for low initial inoculation was already reached after 5 min and 5.4 log CFU/g reduction, whereas for high initial inoculation, the detection limit was reached after 10 min treatment time after a logarithmic reduction of 8.8 log CFU/g.

### Inactivation of *Geobacillus stearothermophilus* Endospores on Lentil Seeds

**FIGURE 6:**
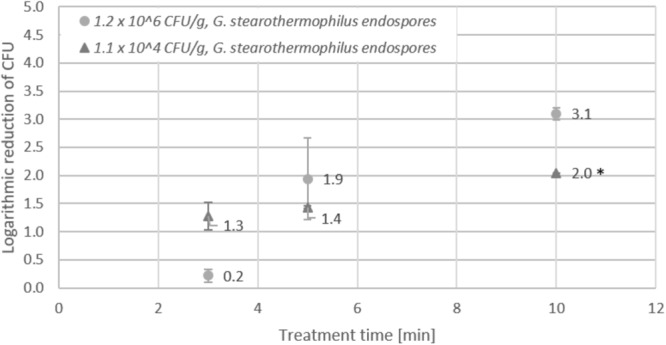
Effect of CAPP treatment of *Geobacillus stearothermophilus* endospores on lentil seeds. Inoculation was performed either as high initial inoculation (1.2 × 10^6^ CFU/g) or low initial inoculation (1.1 × 10^4^ CFU/g) prior to treatment of seeds for 3, 5, or 10 min in the DCSBD. Logarithmic reductions were represented as log10 CFU/g ± standard deviation. The limit of detection for endospores was 100 CFU/g. Logarithmic reduction below the limit of detection is indicated by an asterisk.

Inactivation of *Geobacillus stearothermophilus* endospores on lentil seeds was investigated using a high initial inoculation of 1.2 × 10^6^ CFU/g and a low initial inoculation 1.1 × 10^4^ CFU/g prior to treatment of seeds for 3, 5, or 10 min in the DCSBD. After 3 min treatment, seeds with low initial inoculation revealed a logarithmic reduction of 1.3 log, whereas the logarithmic reduction seeds with high initial inoculation was only 0.2 log (Figure 6). Prolonged treatment of 10 min resulted in an increase of the logarithmic reduction to 3.1 log for seeds with high initial inoculation. For seeds with low initial inoculation, a logarithmic reduction of 2 log was obtained, which represented the detection limit (<100 CFU/g) for low initial inoculation.

### Inactivation of Fungal Spores on Lentil Seeds

**FIGURE 7:**
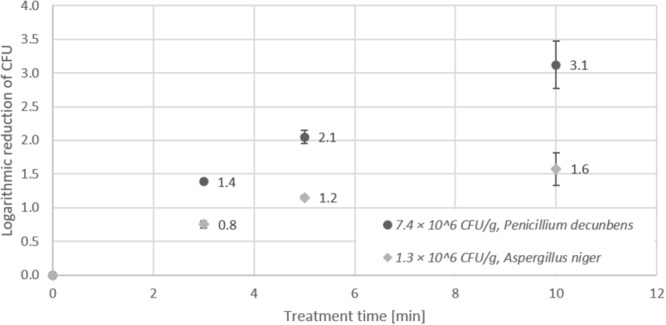
Effect of CAPP treatment of mold spores on lentil seeds. Inoculation was performed either with 7.4 × 10^6^ CFU/g *Penicillium decumbens* spores or 1.3 × 10^6^ CFU/g *Aspergillus niger* spores, prior to treatment of seeds for 3, 5, and 10 min in the DCSBD. Logarithmic reductions were represented as log10 CFU/g ± standard deviation. The limit of detection was 100 CFU/g.

For inactivation of fungal spores, lentil seeds were contaminated with a spore solution prepared either from *Aspergillus niger* or *Penicillium decumbens*. Determination of spore numbers in a microscopic counting chamber revealed 5.5 × 10^7^ spores/ml for the spore solution from *Penicillium decumbens*, and 1.1 × 10^8^ spores/ml for *Aspergillus niger*. After inoculation and drying of the seeds, the recovery was 7.4 × 10^6^ CFU/g for *Penicillium decumbens* and 1.3 × 10^6^ CFU/g for *Aspergillus niger*. In contrast to the inactivation of bacterial pathogens, the logarithmic reduction determined for the fungal spore contaminations was significantly lower and the detection limit (<100 CFU/g) was not reached, even after 10 min treatment in the DCSBD (Figure 7). Inactivation of *Penicillium decumbens* spores on lentil seeds revealed a maximum logarithmic reduction of 3.1 log CFU/g after 10 min treatment, whereas *Aspergillus niger* spores showed a maximum reduction of 1.6 log CFU/g after 10 min.

### Influence of Plasma Treatment on the Germination Capacity of Seeds

**FIGURE 8:**
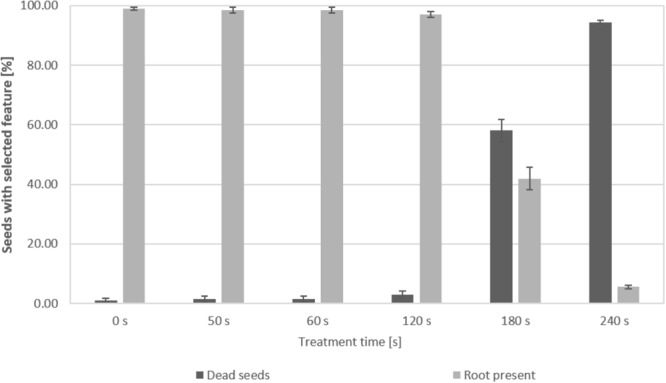
Quantitative assessment of the germination capacity of lentil seedlings after CAPP treatment in the DCSBD. The percentage of dead seeds or germinated seeds, showing the presence of a root, was determined for untreated seeds (0 s) and seeds after increasing plasma exposure times. Error bars express the standard deviation of triplicates for each data point in a 66 seed sample.

The germination capacity was defined as the percentage of lentil seedlings with the presence of a root after 7 days of germination. Figure 8 shows the results obtained for untreated control seeds and seeds treated with an increasing duration of CAPP in the DCSBD. While 120 s treatment time still resulted in 90% germination, the germination capacity dropped to 42% after 180 s exposure of seeds to CAPP. After 240 s CAPP treatment, 95% of the seeds did not germinate.

### Characterization of Mechanisms of Microbial Inactivation by Plasma

Due to the complexity of the plasma multiple components such as UV, temperature, electrons, ion bombardment and reactive species are known to contribute to the antimicrobial effect. In order to improve the understanding of the mechanisms that contribute to the inactivation of microorganisms, an experimental setup was used to selectively investigate the impact of UV and other plasma components on *E. coli* in comparison to untreated bacteria and bacteria treated by whole plasma. After the different treatment regimes, the viability of the bacteria was investigated by performing a CTC stain as an indicator of an intact metabolic activity of the bacterial cells, while a PI stain was used as an indicator of the membrane integrity of the bacteria. Table 2 shows that whole plasma treatment resulted in 81% reduction of viable cells as indicated by a positive CTC stain for 19% of treated cells. Accordingly, 73% of plasma treated cells showed a positive PI stain, which indicated the loss of membrane integrity. Using the experimental setup to shield from UV, only 10 % of bacterial cells showed a PI signal, whereas 74% of treated bacteria were still viable, as indicated by the CTC signal. The lack of CTC signals from *E. coli* cells after selective UV treatment indicated complete inactivation of the bacteria, which was confirmed by a positive PI stain for 99% of the bacteria treated by this experimental setup.

**Table 2:** Percentage of *E. coli* cells showing a fluorescence signal after treatment.

Stain	No treatment	Full plasma	UV	Plasma without UV
CTC-stain	95.2% ± 1.8	19.3% ± 3.2	n.d.	74.1% ± 12.1
PI-stain	0.7% ± 0.7	72.7% ± 12.4	98.6% ± 2.3	10.2% ± 6.4

*n.d., no signal detected.*

### Atomic Force Microscopy Analysis of Plasma Treated Bacteria

**FIGURE 9:**
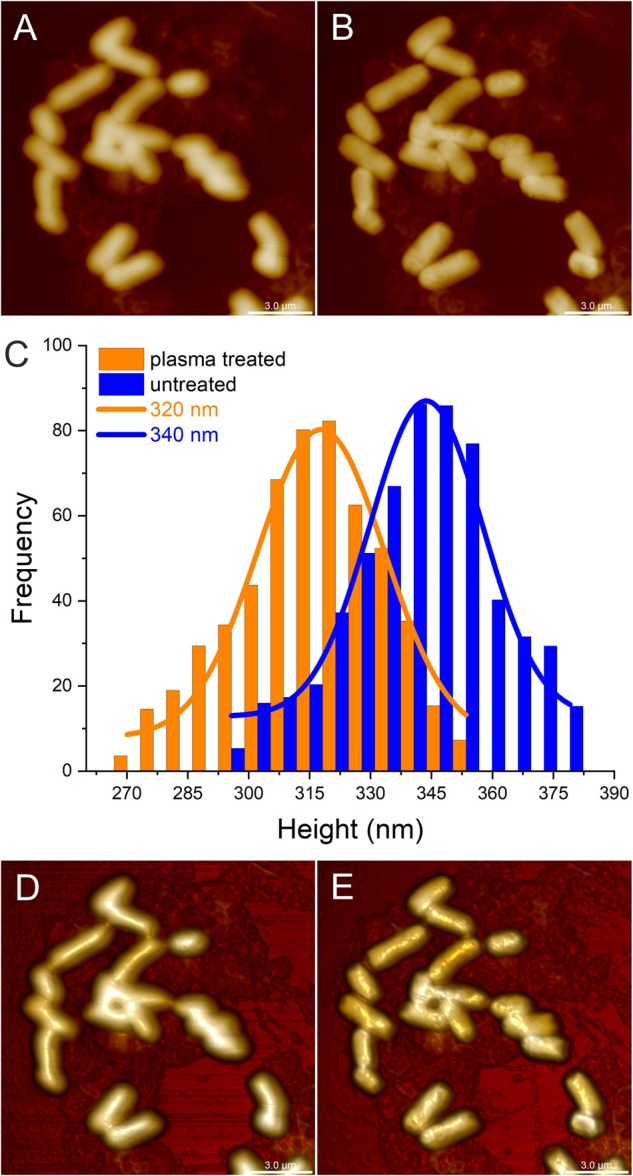
Atomic force microscopy (AFM) imaging of identical *Escherichia coli* cells on a microscopic slide before and after cold atmospheric pressure plasma (CAPP) treatment in the DCSBD system. The upper panel of images represents 2D scans from untreated *E. coli* cells **(A)** and after 1 min **(B)** CAPP treatment as directly generated by AFM. Plot C shows the respective height distribution for untreated *E. coli* cells (blue columns) and *E. coli* cells after 1 min CAPP treatment (orange columns), where the frequency represents the number of *E. coli* cells with a certain height. In comparison to the average height of 340 nm determined for untreated *E. coli* cells (blue line), the average height of *E. coli* cells after 1 min CAPP treatment was decreased by 20 nm resulting in an average height of 320 nm (orange line). The 3-dimensional scans from untreated *E. coli* cells **(D)** and cells after 1 min CAPP treatment **(E)** allow better visualization of the surface of the *E. coli* cells and revealed an increase in the roughness of the cell surface after plasma-treatment. All scale bars represent 3 μm.

Atomic force microscopy was carried out to investigate whether CAPP treatment results in visible changes of the bacterial cell morphology. In order to compare the cell surface topology of identical *E. coli* B174 cells, images were taken from identical positions of the glass slides carrying the immobilized bacteria prior to and after CAPP treatment on the DCSBD system (Figures 9A,B). Treatment of bacteria on the glass slides for 1 min resulted in the reduction of viable *E. coli* below the detection limit, which corresponds to 5 log reduction. In particular, the 3D images shown in Figures 9D,E revealed visible changes in the topography of the cell surface after CAPP treatment, which suggests that there is physical damage to the cell envelope. This observation is further corroborated by the analysis of the height of the bacterial cells deduced from the AFM imaging before and after CAPP treatment (Figure 9C), which revealed a 20 nm decrease in the average height of *E. coli* cells after treatment.

## Discussion

A major goal of this study was to evaluate CAPP generated by a DCSBD system for its suitability as a decontamination technique for seeds used for sprout production. For this reason, the inactivation efficiency for CAPP treatment of relevant foodborne pathogens artificially inoculated on lentil seeds was investigated on a DCSBD system. Besides microbial decontamination, the germination capacity of seeds was assessed after CAPP treatment since it is an important feature for sprout production (NACMCF, 1999). In order to simulate more realistic contamination levels for bacterial pathogens on seeds, a low initial inoculation (10^4^ – 10^5^ CFU/g seeds) was applied in addition to a high initial inoculation (10^8^ – 10^9^ CFU/g seeds), which was necessary to determine the respective maximal logarithmic reduction. The results from the decontamination experiments performed in this study demonstrated an overall high efficiency of CAPP for the inactivation of bacterial pathogens. A maximum logarithmic reduction of 8.8 log after 10 min CAPP treatment was observed for seeds with high initial inoculation of the gram-positive bacteria *Listeria monocytogenes* and *Staphylococcus aureus.* The highest logarithmic reduction of 5.2 log after a shorter CAPP treatment of 3 min was obtained for gram-negative *E. coli*, whereas lentils inoculated with endospores of *Geobacillus stearothermophilus* revealed only low logarithmic reductions between 1 and 2 log after 5 min treatment time.

The results obtained in this study correspond well to the treatment of other granular foodstuffs in similar DCSBD systems. The CAPP treatment of black pepper resulted in logarithmic reductions of >5 log for *E. coli* and *Salmonella*, whereas endospores revealed about 2 log reduction (Mosovska et al., 2018), and the inactivation of *Salmonella* Enteritidis PT30 on the surface of unpeeled almonds by CAPP treatment achieved >5 log reduction of *E. coli* after 15 min treatment of unshelled almonds (Hertwig et al., 2017).

The investigation of the impact of high and low initial inoculation numbers of bacteria on the inactivation efficiency revealed controversial results, which showed a higher logarithmic reduction for treatment of seeds with low initial inoculation for *E. coli* and *Salmonella* Typhimurium (Figures 1, 2). In contrast, higher logarithmic reduction was observed for high initial inoculation with *Listeria monocytogenes* (Figure 3), whereas logarithmic reduction of *Staphylococcus aureus* was not dependent on the initial number of bacteria on the seeds. In a study on the effect of cell surface loading and phase of growth in cold atmospheric gas plasma inactivation of *Escherichia coli* K12, varying numbers of *E. coli* deposited on the surface of membrane filters were exposed to the plume from a cold atmospheric gas plasma (Yu et al., 2006). The authors observed a decrease in reduction by 1 log for higher cell numbers and explained this observation by stacking of bacteria on top of each other in case of high cell numbers (e.g., 9.5 × 10^7^ CFU/ml). Similar results were reported by another study on the application of atmospheric-pressure glow discharges (APGD) for the inactivation of *Bacillus subtilis* endospores, which showed that high microbial loading may lead to a stacking structure that acts as a protective shield against the APGD treatment (Deng et al., 2005). This is in accordance with the CAPP treatment of *Geobacillus stearothermophilus* endospores on lentil seeds in this study, at least for a short treatment time of 3 min. Furthermore, Fernandez et al. (2012) demonstrated that increasing concentrations of *Pseudomonas fluorescens* cells added to a *Salmonella* population of 10^5^ CFU/filter resulted in an exponential decrease in the rate of killing of the *Salmonella* cells. Fluorescence microscopy of the bacteria on polycarbonate membrane filters showed that, unlike single dispersed cells observed at low cell densities, at higher cell densities bacteria were present in a multilayered structure (Fernandez et al., 2012). This stacking phenomenon could explain the reduced inactivation by the plasma, since the top layer may present a physical barrier that protects underlying cells. Since in this study comparable numbers of bacteria were applied for high initial inoculation experiments, it is likely that stacking may have contributed to the lower logarithmic reduction observed for high numbers of *E. coli* and *Salmonella* Typhimurium. A potential explanation for the observation that the stacking effect was less pronounced for *Staphylococcus aureus* might be the fact that staphylococci are much smaller cells with only about half the areal footprint of gram-negative rods. The smaller cells may provide less shielding against the plasma treatment and therefore, the shape and the size of bacterial cells may play a role in the stacking phenomenon, rather than subtle species-dependent cell characteristics. However, although *Listeria* rods are smaller cells compared to rods of *E. coli* and *Salmonella*, this difference hardly explains the contrary effect observed for high inoculation with *Listeria* and *E. coli*.

Due to the known differences in the cell wall structure, it is generally believed that gram-negative bacteria are more sensitive to CAPP than gram-positive bacteria (Hertwig et al., 2018). Consequently, several studies report higher susceptibility of gram-negative bacteria to cold atmospheric air plasma, leading to a more efficient reduction of gram-negative bacteria such as *Salmonella enterica* and *Escherichia coli* compared to gram-positive bacteria such as *Listeria monocytogenes* (Fröhling et al., 2012; Ziuzina et al., 2014). However, this assumption is not supported by the results from this study, which did not indicate a more efficient inactivation of gram-negative bacterial species in general. The logarithmic reduction determined for the gram-positive bacteria *Staphylococcus aureus* and *Listeria monocytogenes* was comparable to gram-negative *Escherichia coli*, whereas gram-negative *Salmonella* Typhimurium turned out to be the least susceptible bacteria tested in this study, at least for high initial inoculation values. This finding is in accordance with the results from a study on the inactivation of foodborne pathogens using atmospheric uniform glow discharge plasma that did not observe differences in the inactivation efficiency for gram-positive and gram-negative bacteria (Kayes et al., 2007). The investigation of cold atmospheric air plasma sterilization against spores and other microorganisms of clinical interest revealed also no indication of a more efficient inactivation of gram-negative bacteria (Klampfl et al., 2012). The effect of 30 s of plasma treatment varied among all tested gram-negative and gram-positive bacteria from about 4–6 log CFU/g reduction. However, fungal cells like *Candida albicans* generally turned out to be more resistant than bacterial cells, and bacterial endospores turned out to be less susceptible than *C. albicans.* This is in accordance to the results from this study, which revealed a higher inactivation efficiency for vegetative cells of bacteria than for fungi and bacterial endospores, which revealed a similar logarithmic reduction using the DCSBD system. Further support for the observed differences in the susceptibility of different microorganisms to CAPP is provided by a study of Lee and colleagues on the sterilization of bacteria, yeast, and bacterial endospores by CAPP, who reported a similar trend of a decreasing efficiency from bacteria to yeast and bacterial endospores (Lee et al., 2006). However, they did not observe a correlation of the inactivation of *B. subtilis* spores with the initial inoculation concentration like it was reported by the aforementioned study from Deng et al. (2005).

Besides the different plasma sterilization systems used for treatments, the observed differences in the efficiency of treatment might be explained by the different substrates that carried the microorganisms, which was flat nitrocellulose membranes compared to lentil seeds used in this study. In the study of Ziuzina et al. (2014), artificially inoculated tomatoes and strawberries were treated with CAPP. The influence of substrate shape and properties on the efficacy of plasma inactivation was already shown in a previous study investigating plasma inactivation of bacterial endospores on wheat grains and polymeric model substrates in a dielectric barrier discharge (Butscher et al., 2016a). Furthermore, it was also shown that moisture content is an important parameter in CAPP treatment that directly impacts the efficiency of CAPP decontamination. Water can trigger a liquid chemistry and may affect the stability of plasma generated bactericidal species, such as reactive oxygen and nitrogen species. On the other hand, it can lead to quenching of the plasma which results in a decrease in plasma intensity as the energy is redirected to the rotational and vibrational excitation of molecules instead of ionization (van Gils et al., 2013; Butscher et al., 2016a).

The lower inactivation efficiency of CAPP treatment for fungi compared to bacteria reported by other studies (Lee et al., 2006; Klampfl et al., 2012) is also consistent with the results from this study. With maximum logarithmic reductions of 1.6 log for *Aspergillus niger* and 3.1 log for *Penicillium decumbens* spores on lentil seeds, the treatment efficiency was significantly lower compared to the inactivation of bacteria, indicating that fungal spores appear to be more resistant to non-thermal plasma. Compared to *Penicillium decumbens*, the values for logarithmic reduction of *Aspergillus niger* spores were approximately cut in half (Figure 7). This observation is controversial to the results from another study on the inactivation of *Aspergillus* spp. and *Penicillium* spp. on seeds using low pressure cold plasma (LPCP), which revealed no significant differences in the inactivation of the two mold species (Selcuk et al., 2008). However, the reported logarithmic reductions of 1 log CFU/g after 5 min and 1.5 log CFU/g after 10 min treatment reported by Selcuk et al. (2008) were in the same low range as in our study for *Aspergillus niger* (Figure 7). Higher resistance of fungal spores to cold plasma compared to bacterial cells is also reported by a recent review on the potential of cold plasma for safe and sustainable food production (Bourke et al., 2018). However, a previously published study on the decontamination of *Aspergillus flavus* and *Aspergillus parasiticus* spores on hazelnuts in an atmospheric pressure fluidized bed plasma reactor revealed significantly higher reductions with 4.5 log CFU/g for *A. flavus* and 4.2 log CFU/g for *A. parasiticus* after 5 min treatment time (Dasan et al., 2016). In contrast to previous studies on the inactivation of fungal spores, more recent work of Zahoranová and colleagues on the effect of CAPP treatment on maize and wheat seeds revealed complete inactivation of 10^6^ spores/g of seeds for *A. flavus* and *A*. *alternata* after 5 min CAPP treatment (Zahoranová, 2016, 2018). Furthermore, these studies confirmed the observation that *Aspergillus* spp. seem to be less susceptible to CAPP treatment than other fungal genera like *Fusarium* or *Penicillium*. The results from these studies demonstrate that although fungal spores seem to be less susceptible to cold plasma treatment than bacteria, the CAPP technology might be adapted accordingly to succeed also in the decontamination of difficult-to-treat target organisms. This was also shown by the successful application of CAPP treatment for the inactivation of bacterial endospores (Butscher et al., 2016b).

In the present study, the heat and desiccation resistant *E. coli* strain B174 strain revealed a slightly lower logarithmic reduction compared to the EHEC surrogate *E. coli* B176. Laroussi and Leipold (2004) concluded that heat has only a small effect on the inactivation of bacteria while reactive oxygen and nitrogen species play the more important role for CAPP treatment in dielectric barrier discharge (DBDs) (Laroussi and Leipold, 2004). However, in a previous study using an in-house developed DBD device for CAPP treatment of seeds (Butscher et al., 2016a), temperature measurements revealed that the environment in which the samples are treated can reach temperatures that may already contribute to an inactivation of microorganisms and at the same time negatively impact the germination capacity of seeds. Furthermore, it was shown that in a DBD plasma, the flow rate of the work gas correlates to the gas temperature, meaning a higher gas flow rate results in an overall cooler plasma (Butscher et al., 2016b). The treatment of seeds artificially inoculated with *E. coli* in the DBD system resulted in logarithmic reductions between 1 and 3 log CFU/g for different types of seeds, with a maximum logarithmic reduction of 3.4 log CFU/g for cress seeds after 10 min treatment time (Butscher et al., 2016a). Thus, the higher reduction rate of 10^5^ CFU/g after 3 min treatment determined for *E. coli* on lentil seeds in this study suggest that the DCSBD system generated a stronger plasma. The assumption that the DCSBD system used in this study produced a stronger CAPP is further supported by the observation of a stronger impact of the CAPP treatment in the DCSBD on the germination capacity of treated seeds. In contrast to a higher germination capacity observed after 3 and 5 min treatment in the DBD plasma (Butscher et al., 2016a), the germination capacity of seeds decreased already after 3 min treatment in the DCSBD plasma applied in this study. A recent study on the application of CAPP for treatment of cucumber and pepper seeds demonstrated that short treatment times preserved or even increased the germination capacity of seeds, whereas for longer treatment times the germination capacity was negatively influenced by heating, ozone and UV radiation produced by the plasma (Štěpánová et al., 2018). High temperatures may also contribute to the observed decrease in the germination capacity after 3 min CAPP treatment in this study. Hence, measures to prevent overheating of seeds, e.g., by mixing on the dielectric plate of the DCSBD, either mechanically or by an airstream should counteract low germination percentage of seeds. Also cooling of the alumina ceramic dielectric plate on which the plasma is generated in future experiments might provide a potential solution for minimizing the detrimental impact of prolonged CAPP treatment on the germination capacity of seeds.

Although we achieved the inactivation of bacteria, endospores, and fungal spores while preserving the germination capacity of seeds when treatment parameters were optimized, the inactivation mechanisms are still poorly understood and this is mainly attributed to the complexity of plasma. CAPP is a reactive cocktail of different components generated by the plasma such as UV photons, charged particles, radicals and other reactive nitrogen, oxygen and hydrogen species, which can act individually and/or synergistically to cause detrimental effects on microorganisms (Hertwig et al., 2018). However, the precise mechanisms of cold atmospheric plasma treatment and the contribution of the single components to the inactivation of microorganisms is still controversially discussed in literature (Lerouge et al., 2000; Boudam et al., 2006; Hertwig et al., 2018).

In order to simplify the investigation of plasma inactivation mechanisms, Schneider and colleagues developed an experimental setup to separate VUV photons from the reactive particles in a plasma jet with Helium gas flow, where neutral particles were pushed through a side channel, allowing only the VUV photons to continue through the direct channel (Schneider et al., 2011). Under the conditions tested, VUV and UV photons alone had only a weak impact on *E. coli* monolayers on agar plates. In contrast, ROS-only and combined treatment revealed that the cells were most probably inactivated by ozone at larger distances from the jet and by a combined effect of ozone, atomic oxygen, and some other possible impurities in the region close to the jet axis. This assumption is further supported by results from a study of Laroussi and Leipold (2004), in which emission spectroscopy and gas detection was used to evaluate important plasma inactivation factors such as UV radiation and reactive species. The measurements indicated that for non-equilibrium, atmospheric pressure air plasmas oxygen-based and nitrogen-based reactive species play the most important role in the inactivation process (Laroussi and Leipold, 2004). Furthermore, microscopic characterization of CAPP inactivation of individual bacterial spores suggested that ROS were the CAPP component causing the most spore killing, with UV-A photons and charged particles being of lesser importance (Wang et al., 2016). To facilitate the selective investigation of the impact of UV and other plasma components (e.g., ozone) on *E. coli* cells, an experimental setup was used to compare their effect with untreated bacteria and bacteria treated by whole plasma in preliminary experiments. For this reason, ozone and UV were measured by optical emission spectroscopy. The results obtained from the separate treatment are summarized in Table 2. Plasma treatment, while shielded from UV, resulted in an inactivation of 26% of the bacterial cells, indicated by the absence of a CTC signal for metabolic activity. Propidium iodide staining of the respective samples revealed a damaged membrane in 10% of treated bacteria. Considering that there was a physical barrier between the plasma and bacterial liquid suspension, our study resulted in an indirect treatment of the bacteria and thus, the distance to the sample likely played a role. With a larger distance, there were probably less reactive species that could reach the bacteria in this setup, whereas UV had a shorter distance to travel in order to reach the sample. Furthermore, although it is commonly known that UV has an effect on DNA, it has been shown that UV affects the membrane integrity in bacteria as well (McKenzie et al., 2016). This might explain the high percentage of bacteria stained by PI after selective UV treatment. On the other hand, an experimental study on the inactivation of bacteria on the surface of agarose gels using dc corona discharge showed that neither UV radiation, ozone or H_2_O_2_ nor other neutral active species alone produced by the corona discharge had an effect on the viability of the bacteria (Dobrynin et al., 2011). Therefore, the strong impact of UV observed in this study (Table 2) might have been favored by the experimental setup and the substrate used in these preliminary experiments. For the selective investigation of UV treatment, the bacteria were applied as liquid suspensions in μ-Slide 2-well uncoated slides covered with a lid to prevent access of ozone and other reactive species. Compared to the more realistic setup of inoculation of bacteria on the seed surface, where the bacteria may be protected from UV radiation by hiding in fissures and cracks on the seed surface (Butscher et al., 2016a), the bacteria in the liquid suspension were readily accessible by the generated UV radiation. This high inactivation was also observed in another study where the treatment of *E. coli* in liquid media inside a sealed package with a dielectric barrier discharge atmospheric cold plasma (DBD-ACP) resulted in the inactivation of high concentrations of *E. coli* in seconds (Ziuzina et al., 2013). Furthermore, in a humid environment, bacterial cells are much more susceptible to elevated temperatures compared to dry conditions, where higher temperatures are less detrimental. This is supported by several studies that investigated dry heat inactivation of bacteria (Beuchat and Scouten, 2002; Bang et al., 2011; Choi et al., 2016; Hong and Kang, 2016). Thus, the experimental setup used for the investigation of isolated CAPP components in this study might have caused an increase in the temperature of the liquid suspension that contributed to the inactivation of the bacteria by membrane damage, as suggested by the high number of bacteria showing a positive PI stain. Additionally, it would be expected that UV would have a different effect on bacterial cells in a liquid suspension compared to the monolayer of bacterial cells on the surface of an agar plate used by the aforementioned study of Schneider et al. (2011). Consequently, further experiments using an improved setup will be necessary in the future to determine the contribution of the different plasma components to the inactivation of microorganisms during CAPP treatment. Not only would an improved setup be needed to better understand the inactivation mechanisms but also the use of different bacteria since the vulnerable cell envelope of gram-negative bacteria might have favored this effect. In a recent study on the inactivation efficiency of in-package high-voltage atmospheric cold plasma (HVACP) and the role of intracellular ROS (Han et al., 2016), the authors reported differences in the mechanisms of inactivation by HVACP observed for different bacterial species. Reactive species were found to either react primarily with the cell envelope or to damage intracellular components. While the inactivation of *E. coli* was mainly due to cell leakage after damage of the cytoplasmic membrane and only low-level DNA damage, the inactivation of *Staphylococcus aureus* cells was primarily due to intracellular damage with significantly higher levels of intracellular ROS observed. However, for both bacteria studied, an increasing treatment time had a positive effect on the intracellular ROS levels generated (Han et al., 2016). Likewise, it was recently shown for *Aspergillus flavus* that CAPP treatment has an impact on cell surface structures, cell wall, and plasma membrane, inflicting injury on hyphal cells that causes oxidative stress and finally cell death at higher CAPP doses (Simoncicova et al., 2018). The results indicated that plasma treatment resulted in accumulation of intracellular ROS and showed that plasma-generated oxidants attack lipids and cause an increase in cell membrane permeability. Furthermore, DNA fragmentation occurred in plasma treated cells.

For further evaluation of the inactivation mechanisms of CAPP on *E. coli*, identical cells were then analyzed by AFM, before and after CAPP treatment. The resulting AFM images revealed distinct morphological alterations in the cell surface topology after plasma exposure as shown for treated *E. coli* cells in the 2D and 3D AFM images (Figure 9). In particular the comparison of 3D AFM images from *E. coli* cells before treatment (Figure 9D) and after treatment (Figure 9E) revealed a rough surface with indentations and swellings all-around the cell surface. The 3D AFM images indicate that the cell surface of the bacteria is considerably affected by the CAPP treatment, showing areas of physical damage distributed over the whole surface. A possible interpretation of this observation might be the partial rupture of the cell wall and release of cytoplasmic content to the outside. This observation was further corroborated by the analysis of the height distribution of *E*. *coli* cells before and after treatment as calculated from the AFM image data (Figure 9C), which revealed a decrease in the average height for *E. coli* cells after CAPP treatment. The observed impact of CAPP treatment on the morphology of the bacterial cells was comparable to other investigations on the impact of plasma treatment on bacteria (Galvin et al., 2013; Cahill et al., 2014). Furthermore, the determination of viable counts after CAPP treatment done in parallel revealed a 5 log reduction after 60 s CAPP treatment, indicating that the bacterial cells recorded after CAPP treatment were dead cells. However, even though the results from the AFM analysis indicate that damage of the cell surface leads to killing of the bacteria, it cannot be undoubtedly concluded that the observed damages are causative for the inactivation of the bacteria. Using Raman spectroscopy and phase-contrast microscopy to characterize CAPP inactivation of bacterial endospores, it was observed that damages observed by microscopy may appear to occur only after spores are already dead due to other causes (Wang et al., 2016). Therefore, the precise mechanisms whereby the spores are killed by CAPP treatment still remains unclear.

Overall, the results from this study indicated that CAPP treatment has the potential for efficient reduction of microorganisms on the seed surface, while preserving the germination properties of seeds, at least for moderate treatment times. The characterization of the inactivation mechanisms by preliminary experiments combining a setup for the separate investigation of CAPP components and confocal laser scanning microscopy analysis indicated that UV radiation is an important component of CAPP, while the application of AFM demonstrated that physical damage of the bacterial cell envelope occurs as a consequence of CAPP treatment of bacterial cells. However, it is important to note that the results obtained in this study provide only preliminary evidence for the role of selective plasma components to the inactivation of microorganisms with CAPP treatment, but the detailed mechanisms of how killing occurs in microorganisms is still not completely clear.

The National Advisory Committee on Microbiological Criteria for Foods (NACMCF) recommends hypochlorite treatment for seeds used for sprouting (NACMCF, 1999). In a study on the decontamination of alfalfa sprouts artificially inoculated with approximately 10^8^ CFU/g *E. coli* by a combined hypochlorite and lactic acid treatment, a reduction of 4 – 6 log CFU/g was achieved after 15 – 25 min treatment, while retaining a high germination capacity of the seeds (Lang et al., 2000). Using CAPP in this study, it was possible to achieve a similar log reduction after a much shorter treatment time of 3 min. However, as discussed by a recent review (Bourke et al., 2018), an improved antimicrobial efficiency due to an increase in treatment duration or input power usually results in an increase of the negative impact on the germination capacity of seeds, which is the most important feature for sprout production. Furthermore, before implementing on an industrial scale, another remaining challenge is the construction of systems that guarantee uniform plasma treatment during continuous processing of seeds. In conclusion, CAPP technology is still under development and has a great potential for further optimization that will allow to maintain the germination capacity of seeds, while providing an effective surface decontamination that contributes to safe and sustainable sprout production.

## Author Contributions

DB, PvR, and MS were responsible for the study conception. MS and DB conceived the experimental design. AW and JB were responsible for data acquisition. DK and AB-M performed sprouting analyses. JA performed the AFM analyses. AW, JB, DB, GO, and MS analyzed and interpreted the data. AW and MS drafted the manuscript. All authors provided critical revisions and approved the manuscript.

## Conflict of Interest Statement

The authors declare that the research was conducted in the absence of any commercial or financial relationships that could be construed as a potential conflict of interest.
